# Diffuse Subcutaneous Emphysema and Pneumomediastinum Secondary to a Minor Blunt Chest Trauma

**DOI:** 10.1155/2017/7589057

**Published:** 2017-03-14

**Authors:** Maximilian Andreas Storz, Eric P. Heymann, Aristomenis K. Exadaktylos

**Affiliations:** ^1^Department of Emergency Medicine, University Hospital of Bern (Inselspital), Bern, Switzerland; ^2^Medical Faculty, Saarland University, Homburg, Germany

## Abstract

Full medical evaluation is paramount for all trauma patients. Minor traumas are often overlooked, as they are thought to bear low injury potential. In this case report, we describe the case of a 48-year-old man presenting to our Emergency Department with mild to moderate right-sided shoulder and scapular pain following a fall from his own height ten days previously. Clinical and paraclinical investigations (CT) revealed diffuse right shoulder pain, with crepitations on palpation of the neck, right shoulder, and right lateral chest wall. Computed tomography (CT) demonstrated right-sided costal fractures (ribs 7 to 9), with diffuse subcutaneous emphysema and pneumomediastinum due to laceration of the visceral and parietal pleura and the adjacent lung parenchyma. In addition, a small ipsilateral pneumothorax was found. Surprisingly, the clinical status was only minimally affected by mild to moderate pain and minor functional impairment.

## 1. Introduction

Pneumomediastinum and subcutaneous emphysema are often a result of spontaneous alveolar wall rupture or, more rarely, of disruption of the upper airways or gastrointestinal tract and are related to the presence of air within the mediastinal cavity or in the subcutaneous tissue, respectively [1–3]. The clinical symptoms of mediastinal and/or subcutaneous emphysema critically depend on the amount of extravasated gas and the degree of extension of the affected areas. Most frequently, they include swelling and crepitus over the involved anatomical site, as well as chest pain, dyspnoea, and dysphagia [1, 4].

Both pneumomediastinum and subcutaneous emphysema are usually diagnosed by conventional chest X-rays; however, noncontrast CT of the chest is more sensitive if only low levels of gas accumulate ‎[5]. Since the presence of subcutaneous cervical and thoracic emphysema is highly suggestive of damage to thoracic structures, the latter examination may also provide further diagnostic benefit.

We present the case of a patient with pneumomediastinum and diffuse subcutaneous emphysema, involving the neck, shoulder, and lateral chest wall following traumatic rib fracture secondary to a minor blunt chest trauma. Although a full clinical examination is normally performed after high velocity impacts, our case report serves as a reminder that this is paramount in all trauma patients, as the extensive radiological findings in our patient remind us that the initial findings correlate poorly with the presenting complaint and history.

## 2. Case Presentation

A 48-year-old man presented to our Emergency Department with a 10-day history of moderate right-sided chest pain. The pain had been constant and stable (5-6/10 on the Visual Analog Scale (VAS) for pain) and radiated into his right arm and scapula. The patient reported an accidental fall on his right chest side prior to the pain, with his shoulder hitting the side of his bed, after slipping on a wet floor. The patient denied any prodrome, head trauma, or loss of consciousness; there were no associated respiratory symptoms, aside from a hoarse cough that became worse since the fall. The pain was initially located on the right side of the chest but progressively radiated into his shoulder and arm over a matter of days. The patient's medical history included insulin-dependent diabetes mellitus, as well as chronic alcohol and nicotine abuse.

Vital parameters revealed a tachycardic patient with a heart rate of 115 beats per minute, blood pressure of 150/110 mmHg, and a respiratory rate of 16 breaths per minute, with SpO_2_ (capillary saturation) of 98% on room air. The patient was afebrile (37.7°C).

Clinically, the patient presented diffuse tissue swelling and crepitus over his right scapula, right shoulder, and chest wall. A small haematoma was noted at the inferior angle of the scapula with tender palpation and reduced range of motion in the right shoulder. Heart sounds were normal, without pathological murmurs. Diffuse wheeze and crepitations were noted bilaterally on both inspiration and expiration.

**Figure 1:**
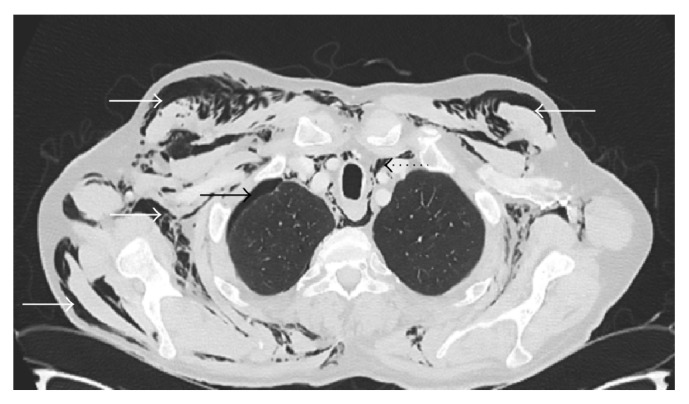
CT revealed diffuse subcutaneous emphysema (white arrows), pneumomediastinum (black dotted arrow), and a small pneumothorax of the right lung (black arrow).

**Figure 2:**
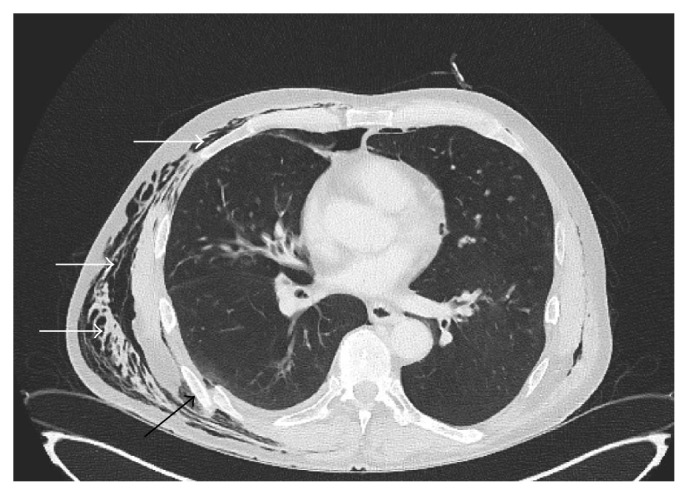
The image shows a displaced fracture of the 7th rib (black arrow). White arrows outline diffuse thoracic subcutaneous emphysema of the lateral chest wall.

**Figure 3:**
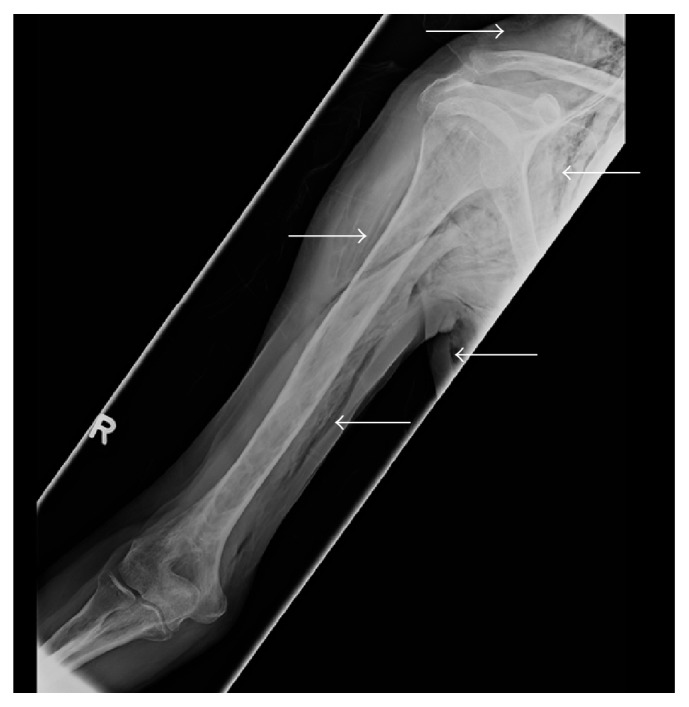
X-ray of the right humeral shaft, indicating emphysema.

Paraclinical investigations revealed mild hyponatraemia (Na: 133 mmol/L; normal: 135–145), with normal leukocyte count (WBC: 7.9 × 10^12^ cells/l; normal: 4.0–10.0 × 10^12^ cells/l), and moderately elevated C-reactive protein (CRP: 37 mg/l; normal: 0–3.3 mg/l); haemoglobin and haematocrit were within the normal ranges. An electrocardiogram (ECG) demonstrated neither ischaemic nor rhythmic pathologies. Chest computed tomography (CT) revealed serial fractures in ribs 7–9 on the right, accompanied by underlying laceration of the visceral and parietal pleura and adjacent lung parenchyma, resulting in extensive subcutaneous emphysema and distinct pneumomediastinum (Figures 1 and 2). Furthermore, a small pneumothorax was noted on the right side (Figure 1). No radiological findings indicating mediastinitis were found on the images. A right humeral shaft fracture was excluded by conventional radiography, which also confirmed subcutaneous emphysema in both upper arm compartments (Figure 3).

**Figure 4:**
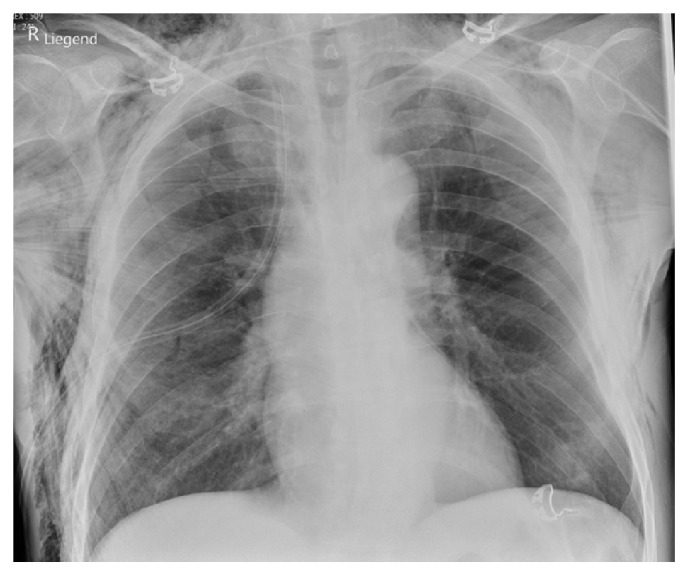
Chest X-ray after insertion of thorax drainage.

The visible and palpable emphysema suggested a bigger pneumothorax and the patient underwent thoracic drainage within our Emergency Department to close the air leak and to avoid further subcutaneous emphysema extension. Furthermore, he received additional analgesic therapy. He was instructed to avoid any strenuous manoeuvres, such as forced expiration or the valsalva manoeuvre, which could potentially lead to increased pulmonary pressure (Figure 4). He was ultimately admitted to our Thoracic Surgery Department for further inpatient management.

## 3. Discussion

Subcutaneous emphysema and pneumomediastinum are rather uncommon clinical entities which usually result from gas leaking from the lungs or other luminal organs. Although pneumomediastinum can occur spontaneously without any known precipitating events, it can also be harbinger to severe injury of mediastinal structures, such as rupture of the oesophagus (Boerhaave syndrome), and should therefore always be further investigated ‎[6]. Traumatic pneumomediastinum was first described by Laennec in 1819 and is the most widely recognized aetiological association ‎[7]. The most commonly accepted explanation for the development of pneumomediastinum is nowadays based on a sequence of events known as the so-called “Macklin Effect” [8, 9]. Once alveolar rupture results, for example, from a primary lung trauma or a constantly increasing high pressure gradient between the alveoli and their surrounding interstitial space, free air dissects along peribronchovascular interstitial sheaths or the visceral pleura. This pulmonary interstitial emphysema finally spreads into the mediastinum [9, 10].

In this specific case, pneumomediastinum was accompanied by diffuse subcutaneous emphysema, due to laceration of the visceral and parietal pleura and the adjacent lung parenchyma. The rib fractures described and shown above most probably lead to laceration of the visceral and parietal pleura and the adjacent lung parenchyma, causing the clinical findings. No antibiotic therapy was given, since subcutaneous emphysema and pneumomediastinum secondary to rib fractures or valsalva maneuver, respectively, are considered benign conditions that resolve spontaneously. Considering the low trauma kinetics and in regard to the fact that the rib fractures sufficiently explain the radiological findings, no thoracoscopy was performed.

What makes this case remarkable are the patient's minimal symptoms and the late presentation, when a graver presenting picture would have been expected. This case serves to emphasize (once more) the importance of a thorough clinical examination of all (trauma) patients and the fact that history must always be followed by a detailed examination (as the initial presentation might have led even the most experienced emergency physician to think of a traumatic shoulder). While CT should be used as a precaution and with good indications, it has proven to be a useful tool in diagnosing pneumomediastinum and quantifying the precise extension of the free air. With careful clinical and paraclinical examinations, we were able to exclude further severe injuries to the mediastinal structures.
